# Gonococcic Urethritis in Boy of 17 Months

**Published:** 1913-11

**Authors:** 


Gonococcic Urethritis in Boy of 17 Months. Archibald Smith and C. S. McKee, *Brit. Med. Jour.,* April 26, 1913, report a case promptly cured by colloid silver. The ætiologic factor was a nurse.

